# A longitudinal study of psychological stress among undergraduate dental students at the University of Jordan

**DOI:** 10.1186/s12909-016-0612-6

**Published:** 2016-03-12

**Authors:** Suha B. Abu-Ghazaleh, Hawazen N. Sonbol, Lamis D. Rajab

**Affiliations:** Department of Pediatric Dentistry and Orthodontics, Faculty of Dentistry, University of Jordan, Amman, Jordan

**Keywords:** Academic environment, Undergraduate dental, Stress, Dental environment stress questionnaire

## Abstract

**Background:**

The purpose of this study was to identify whether psychological stress increased as undergraduate dental students progressed through their studies from first to fifth year. Another objective was to determine if the perceived sources of stress have changed along the years.

**Methods:**

To achieve these aims, a cohort of students at the University of Jordan were followed from first to fifth year of dental school. Fifth year students completed both the General Health Questionnaire ‘GHQ-12’ which was used to assess psychological stress and the Dental Environment Stress questionnaire ‘DES’ which was used to examine the perceived sources of stress. The same cohort of students had completed similar questionnaires during their first year of study. Chi-square analysis and independent t-test analysis were performed to compare GHQ-12 and DES scores between first and fifth year.

**Results:**

Results showed that psychological stress increased from first to fifth year of study. Eighty- nine percent of fifth year students scored over the cut-off point of three in the GHQ-12 compared to 58 % in the first year. The difference between the years was statistically significant at *p* = 0.05. Mean score for DES also increased between first and fifth year of study and the difference was statistically significant at *p* = 0.05.

**Conclusions:**

Results of this study demonstrated that stress in dental students at the University of Jordan increased along the years. Fifth year students showed a high level of psychological stress and methods to reduce that stress should be further investigated and utilized.

## Background

Psychological stress among dental students has been a subject of interest for numerous investigators from around the world [1–18]. Psychological stress occurs when an individual perceives that environmental demands tax or exceed his or her adaptive capacity, resulting in psychological and biological changes that may place the individual at risk for disease [19]. Stress-related symptoms over a long period of time may result in substance abuse and a diminished efficiency at work or learning [12], as well as being negatively related to academic performance and health [14]. Long term exposure to stress has been associated with other psychological problems such as burnout, which can influence mental health [15].

Findings from different studies have indicated that stress in dental students was quite high [1–15]. When compared with students from other health faculties, dental students showed a higher level of stress than medical students [16–18]. Previous studies have predominantly focused on examination of stress in dental students at a certain point of time. Results of these investigations have shown that psychological stress was highest in final year students and lowest in first year students [2, 9, 11]. Some studies however have found that stress was highest in third year students when the transition to clinical teaching occurred [3, 10]. This would seem to imply that as students progressed through dental school, their level of stress increased, but due to the cross-sectional nature of these investigations, this assumption could not be verified.

In a recent systematic review of stress in dental students, it was recommended that more longitudinal investigations be carried out to assess whether stress in dental students actually increased as they advanced through their studies [13]. In one such longitudinal investigation, first year students from four US dental schools were followed over one year and it was found that, measures of the Perceived Stress Scale (PSS) as well as the Dental Environment Stress (DES) scores increased by the end of the year [14]. In another study, psychological stress outcome in fifth year dental students was compared with first year baseline results from five European dental schools and it was found that psychological stress in dental students increased from first to fifth year [15].

The dental school at the University of Jordan offers a five year undergraduate course leading to the acquisition of a Doctor of Dental Surgery (DDS) degree. The first two years are preclinical in content and in the third year, students take an introductory course in clinical dentistry. Fourth and fifth years are clinical years and students are expected to examine and offer treatment to patients under supervision as part of their clinical training.

Psychological stress in dental students at the University of Jordan was assessed in the year 2008 using the General Health Questionnaire (GHQ-12). Results of that cross-sectional study have demonstrated that stress was highest in fifth year students and lowest in first year students [9]. The aim of this investigation was to follow the cohort of first year students who took part in the 2008, cross-sectional study and to reassess the level of psychological stress in their fifth and final year of dental school. Another objective of this investigation was to find out whether the perceived sources of stress have changed between the first and fifth year of study.

## Methods

### Sample description

In accordance with local legislations at The University of Jordan, ethical as well as scientific approval for the study were obtained from the scientific research committee at the Faculty of Dentistry as well as from the Deanship of Academic Research. Data collection took place at the beginning of the second semester of the academic year 2011/2012. Fifth year students enrolled at the Faculty of Dentistry, University of Jordan completed the study questionnaires. This was the same cohort of students that completed similar questionnaires in their first year of dental school during the academic year 2007–2008 [9]. During their first year of dental school, 135 students participated in the study with a response rate of 89 % [9], while 67 students participated during the fifth year of study with a response rate of 50 %.

Students were addressed at a lecture hall following a regular lecture and the purpose of the study was explained by the first author. Students were invited to take part in the study and ensured that participation was voluntary and anonymous. Consent was informed and implicit; if students proceeded to complete the questionnaires following the explanation, it was understood that they consented to participate. All students in attendance at the classroom completed the questionnaires except for one student who opted not to participate. Confidentiality was guaranteed since students were not asked to write their names or any other identifiers such as a university number on the submitted questionnaires.

### Instruments used

Participants were asked to answer three questionnaires in paper and pencil format and it took around 10 min to complete the questionnaires. The first questionnaire collected basic sociodemographic data. This included a question on gender, whether the first choice of university admission was medicine or dentistry, whether students entered university on competitive basis or not, whether they were living at home with their parents or not and whether their education was funded by their parents or not.

The second questionnaire was the General Health Questionnaire ‘GHQ-12’, which was used to assess the psychological distress of students [20]. GHQ-12 is a 12 item questionnaire that asks the respondent to indicate on a Likert-type scale the way they have been feeling over the last four weeks in response to certain questions. An example of the questions is one that is asking the respondent to rate how much they felt that they could not overcome their difficulties in the last four weeks. This was the same questionnaire used in the year 2008 [9]. The validity and reliability of the GHQ-12 was demonstrated previously [21], and its use was recommended for screening of psychological distress in all clinical groups [21]. The GHQ-12 score can be calculated in two ways. The first method is by calculating the average outcome based on a score of 0–3 of the four possible responses to each question [22]. The second method is by using the 0–0–1–1 scoring of the four possible responses, allowing a maximum score of 12 with a cutoff point of more than three. Any student who scores more than three with the 0–0–1–1 scoring method is to be considered as a ‘case’ with evidence of psychological distress [23].

The third questionnaire was the Dental Environment Stress Questionnaire ‘DES’ [24] which measures sources of stress associated with undergraduate dental studies and training. The DES was employed in multiple studies assessing perceived sources of stress in dental students [1–11, 15]. When used with preclinical students, items of the DES relating to clinical situations are omitted [1–11]. The full 38 item questionnaire was used with fifth year students while the shortened, 26 item questionnaire with omission of clinical questions was used with students in their first year. Each question in the DES is scored on a 1–4 basis, with possible answers being (1 = not stressful, 2 = slightly stressful, 3 = moderately stressful, 4 = very stressful). The DES mean score for all questions is then calculated.

### Analytical strategy

Data was entered into SPSS version 20 and analyzed. A missing value analysis was carried out and cases that had considerable missing information (>5 items) were excluded from the analysis. From fifth year data, one case (0.7 %) was excluded and three cases (4.1 %) were excluded from first year data. Following the exclusion of these cases, all variables in the three questionnaires were analyzed in order to calculate the percentage of missing values in each variable.

The percentage of missing values was mostly low; less than 2.5 %, except for the following DES items: ‘amount of cheating at dental school’ 4.5 %, ‘financial resources’ 4 %, ‘lack of confidence of being a successful dentist’ 4 %, and ‘amount of assigned work’ 3 %. Since a missing data proportion of 5 % or less has been considered inconsequential [25], all variables were included in our analysis and multiple imputation was used to substitute for missing values [26].

Descriptive analysis was carried out for the sociodemographic data. Independent t-test analysis was used to compare the two samples from first and fifth year with regards to sociodemographic variables, and no statistically significant differences were found. For the GHQ-12 as well as for the DES, mean, standard deviation and frequency distribution were calculated. The internal consistency of the GHQ-12 and DES was tested with Cronbach’s alpha test. Chi-square analysis and independent t-test analysis were performed to compare GHQ-12 results between first and fifth year and between genders. DES scores were compared between first and fifth year of study using independent t-test analysis. P-value was set at 0.05.

## Results

In total, 67 fifth year students took part in the study, a response rate of 50 %. Following the elimination of one case for missing values, the number dropped to 66. Females constituted 76 % of fifth year sample while 24 % were males. Table 1 gives the breakdown of the sample of fifth year students and compares it with the sample collected when students were in their first year of study. First choice of admission was dentistry for 55 % of fifth year sample and 53 % were admitted through the competitive admission program. Eighty-six percent of fifth year students were living at home with their parents. For 77 % of fifth year students, tuition fees for their dental studies and other related costs were being paid by their parents.

**Table 1:** Demographic details of participating students and response rate

Year of Study	Number of Students	Male	Female	Response Rate
Fifth	66	16	50	49 %
First	132	36	96	87 %

The internal consistency (Cronbach’s alpha) for the GHQ-12 was 0.77, while it was 0.86 for the 26 item DES questionnaire and 0.91 for the full 38 item DES questionnaire. These results indicated that agreement between items in the scales was acceptable, and pointed to the reliability of the scales.

The GHQ-12 mean score using the 0–3 scoring method for students in their first year of studies was 1.2 with standard deviation ‘SD’ =0.5. During their fifth year of study, this cohort of students had a higher mean GHQ-12 score (1.8 with SD = 0.5). The difference between mean scores in first and fifth year was statistically significant (means = 1.2 vs. 1.8, t = 7.3, *p* < 0.0001). Regarding gender differences for fifth year sample, females had a higher mean GHQ-12 score than males which was statistically significant: (means =1.8 vs. 1.4, t = 2.2, *p* < 0.05).

Using the 0–0–1–1 scoring method for GHQ-12 with a cutoff point of more than three, the percentage of students in first year with evidence of psychological distress was 58 %. This increased to 89 % in fifth year, and the difference along the years was statistically significant (percentage of cases = 58 vs. 89 %, *χ*^2^ = 20.5 *p* < 0.0001). ‘Table 2’ When comparing fifth year gender differences with regards to GHQ-12, females had a higher percentage at 91 % while males had a percentage of 75 %. The difference between the sexes was not statistically significant *X*^2^ = 1.9, *p* > 0.05.

**Table 2:** General Health Questionnaire (GHQ-12) by mean (SD), and cases

Year of study	GHQ-12 mean (SD) range 0–3	GHQ-12 cases (score > 3), scale 0–0–1–1 %
Fifth	1.8 (0.5)	89 % (59)
First	1.2 (0.5)	58 % (76)

During their first year of study, this cohort of students had a mean preclinical DES score of 2.2 (0.5). For fifth year students and in order to be able to compare the DES results with those collected during the first year of study; when students had no contact with patients, 12 items relating to clinical situations were omitted from the questionnaire. The mean score for the 26 item DES for fifth year students was 2.8 (SD 0.5). The difference in mean DES score between first and fifth years of study was statistically significant (means = 2.2 vs. 2.8, t = 6.5, *p* < 0.0001) ‘Table 3’.

**Table 3:** Dental Environment Stress Questionnaire (DES) mean scores by year

Year of study	Preclinical DES mean (SD)	Clinical DES mean (SD)
Fifth	2.8 (0.5)	2.9 (0.5)
First	2.2 (0.5)	

The top five stressors in the preclinical DES questionnaire for fifth year students were: ‘a full loaded day’, ‘examination and grades’, ‘amount of assigned work’, ‘late ending time’, and ‘fear of being unable to catch up with work’. They are shown in Table 4 along with the top five stressors during the first year. The means for all the items have increased from first to fifth year. The top stressor for students in their fifth year when omitting clinical items was a ‘full loaded day’. This was different from the top stressor for students in the first year which was ‘examination and grades’.

**Table 4:** Top 5 stressors for the preclinical ‘26 item’ DES

Fifth year results	First year results
Mean (SD)	Mean (SD)
Full loaded day 3.62 (0.7)	Examination and grades 3.23 (0.8)
Examination and grades 3.58 (0.7)	Full loaded day 2.91 (0.9)
Amount of assigned work 3.52 (0.7)	Fear of failing a course or year 2.86 (1.1)
Late ending time 3.49 (0.7)	Lack of relaxation time 2.86 (1.0)
Fear of being unable to catch up with work 3.42 (0.8)	Fear of being unable to catch up with work 2.73 (1.0)

The mean score for the full 38 item DES questionnaire for fifth year students was 2.9 (SD = 0.5). The top five stressors from the 38 item DES questionnaire were: ‘completing clinical requirements’, ‘patients being late or not showing up for their appointments’, ‘full loaded day’, ‘examination and grades’, and ‘amount of assigned work’. The items are shown in Table 5.

**Table 5:** Top 5 stressors for the clinical ‘38 item’ DES

Fifth year top stressors Mean (SD)	
Completing clinical requirements 3.71 (0.6)	
Patients being late 3.63 3.62 (0.7)	
Full loaded day 3.62 (0.7)	
Examination and grades 3.58 (0.7)	
Amount of assigned work 3.52 (0.7)	

## Discussion

Results of the GHQ-12 indicate that stress level has increased with statistical significance between first and final years of study. This is in agreement with other studies that have investigated longitudinal stress in dental students [15], and with other cross sectional studies that have shown that stress level was highest in final year dental students [2, 9, 11]. Dental school appears to be a highly stressful setting with stress level increasing as students progressed through their studies. Medical students however showed a decrease in stress level when GHQ-12 was used to assess stress longitudinally, with the percentage of students with psychological ill health dropping from 37 % in the first year to 22 % in the fifth year [27].

This cohort of students is showing an excessively high level of stress, especially when compared with results from other parts of the world. A study that examined stress in undergraduate dental students from five European dental schools reported the average percentage of fifth year students scoring higher than the cutoff point of three on GHQ-12 was 44 % which is much lower than the percentage reported in this investigation [15]. A previous cross-sectional assessment of stress level in dental students at the University of Jordan however has shown comparable results; with 80 % of fifth year students scoring over the cut-off point of three [9]. The dental school environment especially for final year students at the University of Jordan appears to be highly stressful. During their final year of studies students are expected to accomplish multiple clinical tasks in all specialties as well as successfully pass various theoretical subjects. Further investigations are needed to assess why there is such a high level of stress among dental students at the University of Jordan but another factor that might play a role is high family expectations on performance since most students live with their parents and for most of them, their studies are funded by their parents.

Numerous studies have found a statistically significant difference between genders on the level of stress observed in dental students [3–5, 7–9, 11, 18], although other studies have found no gender effect [1, 6, 15, 17]. Females scored higher than males in our study with regards to stress level and it could be argued that females showed more stress since they were more comfortable in expressing it whereas males especially in our region might be more inclined to hide stress as a show of strength. The difference in stress level between genders was not consistently statistically significant in this study and it could be due to the fact that we did not have enough power in the sample to detect differences between males and females.

There was a statistically significant increase along the years in the mean score for the preclinical 26 item DES questionnaire. This was in agreement with other cross-sectional studies that have found that the highest DES score was reported by final year students [2, 9, 11]. In a longitudinal study that followed undergraduate students from five European dental schools, no difference in the percentage of high scorers on the DES was found between first and fifth year of study, but the authors found differences among the dental schools themselves, with three of the five schools showing an increase while two schools showed a decrease in the percentage of DES high scorers [15].

Results of the DES questionnaire might shed some light into what students regard as a cause of stress in the dental school setting. In this investigation, the top perceived sources of stress when considering the preclinical items changed a little between first and fifth years of study, although the mean score for each of the stressors increased. In their fifth year of study, the top stressor for this cohort of students was ‘a full loaded day’, whereas it was ‘examination and grades’ in their first year of dental school. During fifth year, students had multiple clinics and lab work that took up much more time than the mostly theoretical courses they had in their first year. Another item that appeared among the top five stressors for fifth year students was ‘late ending time’, when it was not a major concern for first year students. Having a full loaded day and a late ending time among the top perceived sources of stress showed that students had a lot of work to do in their fifth year of study and that this was taking up a considerable amount of their time.

The above might offer partial explanation for the level of high stress that fifth year students expressed since they had little time for recreational activities that could help in relieving stress. Programs such as yoga, humor and reading have been shown to decrease stress among students in the health sciences [28]. Encouraging students to partake in these simple interventions might help decrease the high level of stress observed. An attempt can also be made to lessen the time students spend at the dental school during the week or else arrange their program in such a way so that they are given some time off during the week.

When considering the full clinical DES questionnaire the top perceived source of stress for fifth year students was ‘completing clinical requirements’. This item was also among the top perceived source of stress for fifth year students in previous examinations of stress in dental students at the University of Jordan [2, 9]. With such a high level of psychological distress reported by fifth year students and with completing clinical requirements consistently being among the top perceived sources of stress, it is hoped that with the recent gradual move to a competency based assessment of clinical courses, this would lessen the emphasis on the number of clinical requirements that has to be completed and reduce the level of stress exhibited by students. Competency based assessment was started at the University of Jordan during the academic year 2013–2014. In a preliminary study by Dodge et al., it was found that improvement in clinical performance as well as a reduction in stress level was observed in students who completed a clinical program based on patient needs rather than clinical requirements [29].

To help students cope with the stress of completing their undergraduate dental studies, a recent systematic review of stress management in dental students recommended the introduction of stress management programs as part of the dental curriculum, although the nature of such programs needed further investigation [30]. Examples of stress management programs were exercises such as deep breathing and muscle relaxation techniques [31]. Yoga exercises were effective in reducing stress in dental students performing their first periodontal surgery [32]. Peer mentoring has been shown to help dental students cope better with stress [33] and would be an easily implemented approach to aid students throughout their dental education.

### Limitations

Students were asked to answer the questionnaires anonymously. Anonymity might have helped in getting true representation of how a student felt about items in the questionnaires but it was limiting in that it was not possible to follow up with the same student from first to fifth year. Students can differ in their inherent anxiety and ability to cope with stressful situations [34], therefore it might be better in the future when investigating stress longitudinally to take that into consideration.

## Conclusions

Psychological stress level increased as dental students progressed from first to fifth year of study. Fifth year dental students at the University of Jordan had a high level of stress when compared to results from other parts of the world. Programs to reduce that level of stress need to be further investigated and implemented.

## Abbreviations

GHQ-12: general health questionnaire
DES: dental environment stress questionnaire
SD: standard deviation

## References

[1] Humphris G, Blinkhorn A, Freeman R, Gorter R, Hoad-Reddick G, Murtomaa H (2002). Psychological stress in undergraduate dental students: baseline results from seven European dental schools. Eur J Dent Educ.

[2] Rajab LD (2001). Perceived sources of stress among dental students at the University of Jordan. J Dent Educ.

[3] Naidu RS, Adams JS, Simeon D, Persad S (2002). Sources of stress and psychological disturbance among dental students in the West Indies. J Dent Educ.

[4] Sugiura G, Shinada K, Kawaguchi Y (2005). Psychological well-being and perceptions of stress amongst Japanese dental students. Eur J Dent Educ.

[5] Polychronopoulou A, Divaris K (2005). Perceived sources of stress among Greek dental students. J Dent Educ.

[6] Sofola OO, Jeboda SO (2006). Perceived sources of stress in Nigerian dental students. Eur J Dent Educ.

[7] Peker I, Alkurt MT, Usta MG, Turkbay T (2009). The evaluation of perceived sources of stress and stress levels among Turkish dental students. Int Dent J.

[8] Polychronopoulou A, Divaris K (2009). Dental students’ perceived sources of stress: a multi-country study. J Dent Educ.

[9] Abu-Ghazaleh SB, Rajab LD, Sonbol HN (2011). Psychological stress among dental students at the University of Jordan. J Dent Educ.

[10] Alzahem AM, Van der Molen HT, De Boer BJ (2013). Effect of year of study on stress levels in male undergraduate dental students. Adv Med Educ Pract.

[11] Uraz A, Tocak YS, Yozgatligil C, Cetiner S, Bal B (2013). Psychological well-being, health, and stress sources in Turkish dental students. J Dent Educ.

[12] Alzahem AM, van der Molen HT, Alaujan AH, Schmidt HG, Zamakhshary MH (2011). Stress amongst dental students: a systematic review. Eur J Dent Educ.

[13] Elani HW, Allison PJ, Kumar RA, Mancini L, Lambrou A, Bedos C (2014). A systematic review of stress in dental students. J Dent Educ.

[14] Silverstein ST, Kritz-Silverstein D (2010). A longitudinal study of stress in first-year dental students. J Dent Educ.

[15] Gorter R, Freeman R, Hammen S, Murtomaa H, Blinkhorn A, Humphris G (2008). Psychological stress and health in undergraduate dental students: fifth year outcomes compared with first year baseline results from five European dental schools. Eur J Dent Educ.

[16] Schmitter M, Liedl M, Beck J, Rammelsberg P (2008). Chronic stress in medical and dental education. Med Teach.

[17] Birks Y, McKendree J, Watt I (2009). Emotional intelligence and perceived stress in health care students: a multi-institutional, multi-professional survey. BMC Med Educ.

[18] Waghachavare VB, Dhumale GB, Kadam YR, Gore AD (2013). A Study of Stress among Students of Professional Colleges from an Urban area in India. Sultan Qaboos Univ Med J.

[19] Cohen S, Kessler RC, Gordon UL, Cohen S, Kessler RC, Gordon UL (1995). Strategies for measuring stress in studies of psychiatric and physical disorder. Measuring Stress: A Guide for Health and Social Scientists.

[20] Goldberg D, Williams P (1988). A user’s guide to the general health questionnaire.

[21] Banks MH (1983). Validation of the general health questionnaire in a young community sample. Psychol Med.

[22] Goldberg DP, Gater R, Sartorius N, Ustun TB, Piccinelli M, Gureje O (1997). The validity of two versions of the GHQ in the WHO study of mental illness in general health care. Psychol Med.

[23] Guthrie EA, Black D, Shaw CM, Hamilton J, Creed FH, Tomenson B (1995). Embarking upon a medical career: psychological morbidity in first year medical students. Med Educ.

[24] Grandy TG, Westerman GH, Mitchell RE, Lupo JV (1984). Stress among first-year dental students. J Dent Educ.

[25] Schafer JL (1999). Multiple imputation: a primer. Stat Methods Med Res.

[26] Dong Y, Peng CY (2013). Principled missing data methods for researchers. Springerplus.

[27] Guthrie E, Black D, Bagalkote H, Shaw C, Campbell M, Creed F (1998). Psychological stress and burnout in medical students: a five-year prospective longitudinal study. J R Soc Med.

[28] Denise R, Genevieve Pinto Z, Doreen S, Susan S (2009). Stress management strategies for students: The immediate effects of yoga, humor, and reading on stress. J Coll Teach Learn.

[29] Dodge WW, Dale RA, Hendricson WD (1993). A preliminary study of the effect of eliminating requirements on clinical performance. J Dent Educ.

[30] Alzahem AM, Van der Molen HT, Alaujan AH, De Boer BJ (2014). Stress management in dental students: a systematic review. Adv Med Educ Pract.

[31] Piazza-Waggoner CA, Cohen LL, Kohli K, Taylor BK (2003). Stress management for dental students performing their first pediatric restorative procedure. J Dent Educ.

[32] Shankarapillai R, Nair MA, George R (2012). The effect of yoga in stress reduction for dental students performing their first periodontal surgery: a randomized controlled study. Int J Yoga.

[33] Lopez N, Johnson S, Black N (2010). Does peer mentoring work? Dental students assess its benefits as an adaptive coping strategy. J Dent Educ.

[34] Dahan H, Bedos C (2010). A typology of dental students according to their experience of stress: a qualitative study. J Dent Educ.

